# Practical Notes on Diagnosis and Treatment

**Published:** 1906-07-14

**Authors:** 


### Practical Notes on Diagnosis and Treatment.

**Treatment in Hæmatemesis.**

For severe hæmatemesis the immediate treatment is in the first place the giving of ice to suck, secondly the injection of morphine, and thirdly the administration by the mouth of adrenalin.—*Dr. W. P. Herringham.*

**Formula for Mouth Wash.**

A pleasant and efficacious mouth wash, which not only hardens the gums but keeps the mouth sweet, is the following: Lead acetate, 2½ drams; alum trisulphate, 5 drams; boiling water, 20 ounces. Dissolve the salts separately, mix, filter, and to the clear filtrate add essence of peppermint half a dram. *Mr. Campbell Williams.*

**Adrenalin in Pernicious Anæmia.**

Dr. S. West describes a case of pernicious anæmia in which, though meat juice, raw meat, and bone marrow were being administered, no improvement took place until adrenalin solution (1 in 1,000) was ordered. Great improvement then occurred, the red corpuscles being increased to the extent of 50 per cent. The dose was 20 minims thrice daily.—*Clinical Journal,* May 30, 1906.

**Local Measures in Phlebitis.**

These should be directed to relieving pain and insuring rest to the affected limb. For the former thin flannel bags filled with bran and heated in an oven are convenient; they are light and retain heat for a long time. When only a small portion of a vein is involved a hot lead and opium lotion, or a fomentation of boric lint covered by jaconet, is comfortable; but the extensive use of wet applications is inconvenient and involves frequent disturbance. When pain is not severe, the most comfortable dressing is a layer of Gamgee tissue, secured round the limb by a many-tailed bandage, the skin having first been dusted over with powdered boric acid. If the inflamed vein is superficial and tender, it is well to paint along the line of redness a thick lotion of oxide of zinc, glycerine, and carbolic acid in water.—*Mr. Warrington Haward.*

**Progressive Muscular Atrophy.**

Massage and the hypodermic injection of strychnine are the most hopeful therapeutic measures, but the prognosis, even at the best, is an unhopeful one.

**Tabes Dorsalis and General Paralysis.**

"I would express my belief in the essential pathological identity of tabes and general paralysis. They are, in my opinion, merely different aspects of the same polymorphic disease; . . . the morbid process underlying both is identical."—*Dr. David Ferrier.*

